# A New Pulse Coupled Neural Network (PCNN) for Brain Medical Image Fusion Empowered by Shuffled Frog Leaping Algorithm

**DOI:** 10.3389/fnins.2019.00210

**Published:** 2019-03-20

**Authors:** Chenxi Huang, Ganxun Tian, Yisha Lan, Yonghong Peng, E. Y. K. Ng, Yongtao Hao, Yongqiang Cheng, Wenliang Che

**Affiliations:** ^1^Department of Computer Science and Technology, Tongji University, Shanghai, China; ^2^Faculty of Computer Science, University of Sunderland, Sunderland, United Kingdom; ^3^School of Mechanical and Aerospace Engineering, Nanyang Technological University, Singapore, Singapore; ^4^School of Engineering and Computer Science, University of Hull, Kingston upon Hull, United Kingdom; ^5^Department of Cardiology, Shanghai Tenth People’s Hospital, Tongji University School of Medicine, Shanghai, China

**Keywords:** single-photon emission computed tomography image, computed tomography image, image fusion, pulse coupled neural network, shuffled frog leaping

## Abstract

Recent research has reported the application of image fusion technologies in medical images in a wide range of aspects, such as in the diagnosis of brain diseases, the detection of glioma and the diagnosis of Alzheimer’s disease. In our study, a new fusion method based on the combination of the shuffled frog leaping algorithm (SFLA) and the pulse coupled neural network (PCNN) is proposed for the fusion of SPECT and CT images to improve the quality of fused brain images. First, the intensity-hue-saturation (IHS) of a SPECT and CT image are decomposed using a non-subsampled contourlet transform (NSCT) independently, where both low-frequency and high-frequency images, using NSCT, are obtained. We then used the combined SFLA and PCNN to fuse the high-frequency sub-band images and low-frequency images. The SFLA is considered to optimize the PCNN network parameters. Finally, the fused image was produced from the reversed NSCT and reversed IHS transforms. We evaluated our algorithms against standard deviation (SD), mean gradient (Ḡ), spatial frequency (SF) and information entropy (E) using three different sets of brain images. The experimental results demonstrated the superior performance of the proposed fusion method to enhance both precision and spatial resolution significantly.

## Introduction

In 1895 Rontgen obtained the first human medical image by X-ray, after which research of medical images gained momentum, laying the foundation for medical image fusion. With the development of both medical imaging technology and hardware facilities, a series of medical images with different characteristics and information were obtained, contributing to a key source of information for disease diagnosis. At present, clinical medical images mainly include Computed Tomography (CT) images, Magnetic Resonance Imaging (MRI) images, Single-Photon Emission Computed Tomography (SPECT) images, Dynamic Single-Photon Emission Computed Tomography (DSPECT) and ultrasonic images, etc. (Jodoin et al., 2015; Hansen et al., 2017; Zhang J. et al., 2017). It is necessary to fuse different modes of medical images into more informative images based on fusion algorithms, in order to provide doctors with more reliable information during clinical diagnosis (Kavitha and Chellamuthu, 2014; Zeng et al., 2014). At present, medical image fusion has been considered in many aspects, such as the localization of brain diseases, the detection of glioma, the diagnosis of AD (Alzheimer’s disease), etc. (Huang, 1996; Singh et al., 2015; Zeng et al., 2018).

Image fusion is the synthesis of images into a new image using a specific algorithm. The space-time relativity and complementarity of information in fused images can be fully used in the process of image fusion, contributing to a more comprehensive expression of the scene (Wu et al., 2005; Choi, 2006). Conventional methods of SPECT and CT fusion images mainly include component substitution and multi-resolution analysis (Amolins et al., 2007; Huang and Du, 2008; Huang and Jiang, 2012). Component substitution mainly refers to intensity-hue-saturation (IHS) transform, with the advantage of improving the spatial resolution of SPECT images (Huang, 1999; Rahmani et al., 2010). The limitation of transform invariance leads to difficulty in extracting both image contour and edge details. In order to solve this problem, contourlet transform was proposed by Da et al. (2006), Zhao et al. (2012), Xin and Deng (2013). Moreover, non-subsampled contourlet transform (NSCT) was also proposed to fully extract the directional information of SPECT images and CT images to be fused, providing better performance in image decomposition (Da et al., 2006; Wang and Zhou, 2010; Yang et al., 2016).

The Pulse Coupled Neural Network (PCNN) was discovered by Eckhorn et al. (1989) in the 1990s while studying the imaging mechanisms of the visual cortex of small mammals. No training process is required in the PCNN and useful information can be obtained from a complex background through the PCNN. Nevertheless, the PCNN has its shortcomings, such as the numerous parameters and the complicated process of setting parameters. Thus, novel algorithms to optimize the PCNN parameters has been introduced to improve the calculation speed of PCNN (Huang, 2004; Huang et al., 2004; Jiang et al., 2014; Xiang et al., 2015). SFLA is a new heuristic algorithm first presented by Eusuff and Lansey, which combines the advantages of the memetic algorithm and particle swarm optimization. The algorithm can search and analyze the optimal value in a complex space with fewer parameters and has a higher performance and robustness (Samuel and Asir Rajan, 2015; Sapkheyli et al., 2015; Kaur and Mehta, 2017).

In our study, a new fusion approach based on the SFLA and PCNN is proposed to address the limitations discussed above. Our proposed method not only innovatively uses SFLA optimization to effectively learn the PCNN parameters, but also produces high quality fused images. A series of contrasting experiments are discussed in view of image quality and objective evaluations.

The remaining part of the paper is organized as follows. Related work is introduced in Section “Related Works.” The fusion method is proposed in Section “Materials and Methods.” The experimental results are presented in Sections “Result” and “Conclusion” concludes the paper with an outlook on future work.

## Related Works

Image fusion involves a wide range of disciplines and can be classified under the category of information fusion, where a series of methods have been presented. A novel fusion method, for multi-scale images has been presented by Zhang X. et al. (2017) using Empirical Wavelet Transform (EWT). In the proposed method, simultaneous empirical wavelet transforms (SEWT) were used for one-dimensional and two-dimensional signals, to ensure the optimal wavelets for processed signals. A satisfying visual perception was achieved through a series of experiments and in terms of objective evaluations, it was demonstrated that the method was superior to other traditional algorithms. However, time consumption of the proposed method is high, mainly during the process of image decomposition, causing application difficulties in a real time system. Noised images should also be considered in future work where the process of generating optimal wavelets may be affected (Zeng et al., 2016b; Zhang X. et al., 2017).

Aishwarya and Thangammal (2017) also proposed a fusion method based on a supervised dictionary learning approach. During the dictionary training, in order to reduce the number of input patches, gradient information was first obtained for every patch in the training set. Second, both the information content and edge strength was measured for each gradient patch. Finally, the patches with better focus features were selected by a selection rule, to train the over complete dictionary. Additionally, in the process of fusion, the globally learned dictionary was used to achieve better visual quality. Nevertheless, high computational costs also exist in this proposed approach during the process of sparse coding and final fusion performance, which may be affected by high frequency noise (Zeng et al., 2016a; Aishwarya and Thangammal, 2017).

Moreover, an algorithm for the fusion of thermal and visual images was introduced by M Kanmani et al. in order to obtain a single comprehensive fused image. A novel method called self tuning particle swarm optimization (STPSO) was presented to calculate the optimal weights. A weighted averaging fusion rule was also used to fuse the low frequency- and high frequency coefficients, obtained through Dual Tree Discrete Wavelet Transform (DT-DWT) (Kanmani and Narasimhan, 2017; Zeng et al., 2017a). Xinxia Ji et al. proposed a new fusion algorithm based on an adaptive weighted method in combination with the idea of fuzzy theory. In the algorithm, a membership function with fuzzy logic variables were designed to achieve the transformation of different leveled coefficients by different weights. Experimental results indicated that the proposed algorithm outperformed existing algorithms in aspects of visual quality and objective measures (Ji and Zhang, 2017; Zeng et al., 2017b).

## Materials and Methods

### The Image Fusion Method Based on PCNN and SFLA

The algorithm 3.1 represents an image fusion algorithm based on the PCNN and SFLA, where SPECT and CT images are fused. In our proposed algorithm, a SPECT image is first decomposed on three components using IHS transform, which include saturation S, hue H and intensity I. Component I is then decomposed to a low-frequency and high-frequency image through NSCT decomposition. Additionally, a CT image is decomposed into a low-frequency and high-frequency image through NSCT decomposition. Moreover, the two low-frequency images obtained above are fused in a new low-frequency image through the SFLA and PCNN combination fusion rules, while the two high-frequency images obtained above are fused into a new high-frequency image through the SFLA and PCNN combination fusion rules. Next, the new low-frequency and new high-frequency images are fused to generate a new image with intensity I’ using reversed NSCT. Finally, the target image is achieved by using reversed IHS transform to integrate the three components S, H and I’.

**Table:** 

Algorithm 1: An image fusion algorithm based on PCNN and SFLA
Input: A SPECT image A and a CT image B
Output: A fused image F
Step 1: Obtain three components of image A using IHS transform; saturation S, hue H and intensity I.
Step 2: Image decomposition
(1) Decompose the component I of image A to a low-frequency image AL and high-frequency image AH through NSCT decomposition.
(2) Decompose image B to a low-frequency image BL and high-frequency image BH through NSCT decomposition.
Step 3: Image fusion
(1) Fuse the low-frequency images AL and BL to a new low-frequency image CL through the SFLA and PCNN combination fusion rules.
(2) Fuse the high-frequency images AH and BH to form a new high-frequency image CH through the SFLA and PCNN combination fusion rules.
Step 4: Inverse transform
Fuse the low-frequency image CL and high-frequency image CH to a new image with intensity I’ using reversed NSCT.
Step 5: Reversed IHS transform
Through the reversed IHS transform, integrate the three components S, H and I’, then obtain the target image F.

**FIGURE 1:**
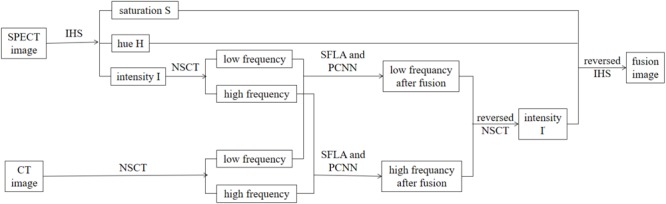
The proposed method for the process of fusion.

The overall method of the proposed algorithm for the fusion of a SPECT and CT image is outlined in Figure 1.

### Decomposition Rule

In our proposed method, the SPECT image and CT image are decomposed into a low-frequency and high-frequency image using NSCT.

**FIGURE 2:**
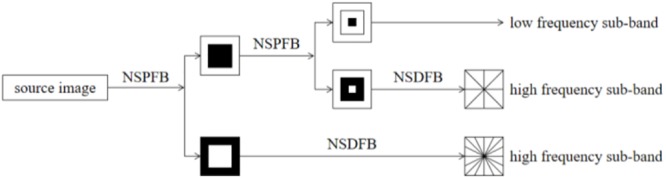
The structure diagram of the two-level decomposition of NSCT.

Non-subsampled contourlet transform (Huang, 1999; Rahmani et al., 2010) is composed of a non-subsampled pyramid filter bank (NSPFB) and a non-subsampled directional filter bank (NSDFB). The source image is decomposed into a high-frequency sub-band and a low-frequency sub-band by NSPFB. The high-frequency sub-band is then decomposed into a sub-band of each direction by NSDFB. The structure diagram of the two-level decomposition of NSCT is shown in Figure 2.

An analysis filter {H_1_ (z), H_2_ (z)} and a synthesis filter {G_1_ (z), G_2_ (z)} are used when using NSCT to decompose images and the two filters satisfy H_1_(z)G_1_(z) + H_2_(z)G_2_(z) = 1. The source image can generate low-frequency and high-frequency sub-band images when it is decomposed by NSP. The next level of NSP decomposition is performed on low-frequency components obtained by the upper-level decomposition. An analysis filter {U_1_ (z), U_2_ (z)} and synthesis filters {V_1_ (z), V_2_ (z)} are contained in the design structure of NSDFB with the requirement of U_1_(z)V _1_(z) + U_2_(z)V _2_(z) = 1. The high-pass sub-band image decomposed by J-level NSP is decomposed by L-level NSDFB, and the high-frequency sub-band coefficients can be obtained at the number of 2^n^, where n is an integer higher than 0. A fused image with clearer contours and translation invariants can be obtained through the fusion method based on NSCT (Xin and Deng, 2013).

### Fusion Rule

Fusion rules affect image performance, so the selection of fusion rules largely determines the quality of the final fused image. In this section, the PCNN fusion algorithm based on SFLA is introduced for low-frequency and high-frequency sub-band images decomposed by NSCT.

#### Pulse Coupled Neural Network

The PCNN is a neural network model of single-cortex feedback, to simulate the processing mechanism of visual signals in the cerebral cortex of cats. It consists of several neurons connected to each other, where each neuron is composed of three parts: the receiving domain, the coupled linking modulation domain and the pulse generator. In image fusion using the PCNN, the M ^∗^ N neurons of a two-dimensional PCNN network correspond to the M ^∗^ N pixels of the two-dimensional input image, and the gray value of the pixel is taken as the external stimulus of the network neuron. Initially, the internal activation of neurons is equal to the external stimulation. When the external stimulus is greater than the threshold value, a natural ignition will occur. When a neuron ignites, its threshold will increase sharply and then decay exponentially with time. When the threshold attenuates to less than the corresponding internal activation, the neuron will ignite again, and the neuron will generate a pulse sequence signal. The ignited neurons stimulate the ignition of adjacent neurons by interacting with adjacent neurons, thereby generating an automatic wave in the activation region to propagate outward (Ge et al., 2009).

The parameters of the PCNN affect the quality of image fusion, and most current research uses the method of regressively exploring the values of parameters, which is subjective to a certain degree. Therefore, how to reasonably set the parameters of the PCNN is the key to improving its performance. In our paper, SFLA is used to optimize the PCNN network parameters.

#### Shuffled Frog Leaping Algorithm

**FIGURE 3:**
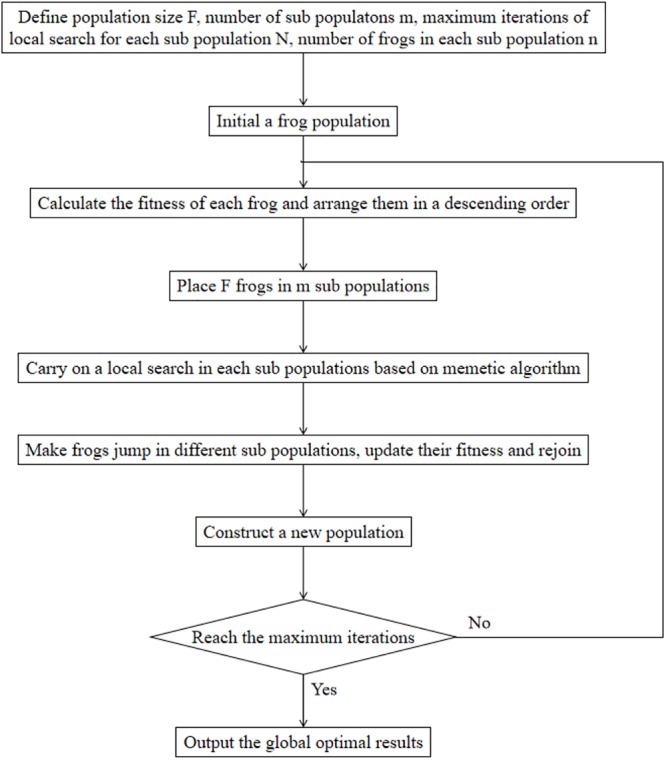
The flowchart of the shuffled frog leaping algorithm.

Shuffled frog leaping algorithm is a particle swarm search method based on groups to obtain optimal results. The flowchart of SFLA is shown in Figure 3. First, the population size F, the number of sub populations m, the maximum iterations of local search for each sub population N and the number of frogs in each sub population n were defined. Second, a population was initialed, and the fitness value of each frog was calculated and sorted in a descending order. A memetic algorithm is used in the process of the search, and the search is carried out in groups. All groups are then fused, and the frogs are sorted according to an established rule. Moreover, the frog population is divided based on the established rules, and the overall information exchange is achieved using this method until the number of iterations are equal to the maximum iterations N (Li et al., 2018).

F(x) is defined as a fitness function and Ω is a feasible domain. In each iteration, P_g_ is the best frog for a frog population, P_b_ represents the best frog for each group and P_w_ is the worst frog for each group. The algorithm adopts the following update strategy to carry out a local search in each group:

(1) $$\begin{array}{c} \{\begin{array}{l} S_{j}=rand()\cdot (P_{b}-P_{w}),\;-S_{\max}\leq S_{j}\leq S_{\max}\\ P_{w,\mathrm{new}}=P_{w}+S_{j} \end{array} \end{array}$$

where S_j_ represents the updated value of frog leaping, rand () is defined as the random number between 0 and 1, S_max_ is described as the maximum leaping value, and P_w,new_ is the worst frog of updated group. If P_w,new_ ∈ Ω and F(P_w,new_) > F(P_w_), P_w_ can be replaced by P_w,new_, otherwise, P_b_ will be replaced by P_g_. At the same time, if P′_w,new_ ∈ Ω and F(P′_w,new_) > F(P_w_), P_w_ can be replaced by P′_w,new_, otherwise P_w_ can be replaced by a new frog and then the process of iteration will continue until the maximum iterations is reached.

#### PCNN Fusion Algorithm Based on SFLA

**FIGURE 4:**
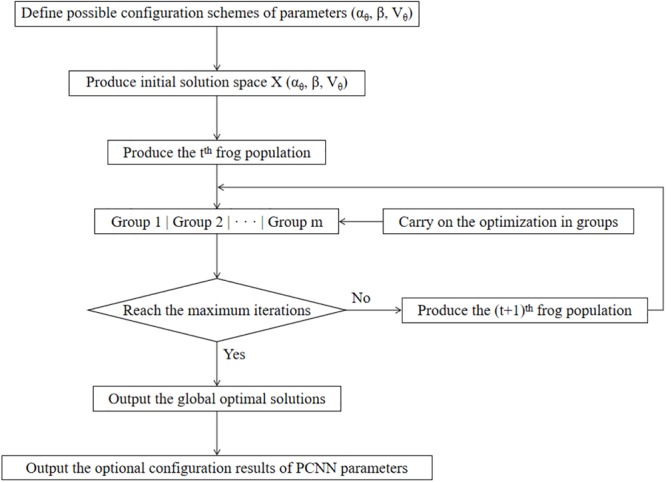
The process of PCNN parameter optimization based on SFLA.

Three parameters α_𝜃_,β and V _𝜃_ in PCNN are essential for the results of image fusion. Therefore, as it is shown in Figure 4, in our study, the SFLA is used to optimize the PCNN in order to achieve the optimal solution of the PCNN parameters. Each frog is defined as a spatial solution X(α_𝜃_,β,V _𝜃_) and the optimal configuration scheme of the PCNN parameters can finally be obtained by searching for the best frog X_b_(α_𝜃_,β,V _𝜃_).

In our proposed method, possible configuration schemes of parameters are defined, which constitute a solution space for the parameter optimization. After generating an initial frog solution space, F frogs in the population are divided into m groups, and each group is dependent on one another. Starting from the initial solution, the frogs in each group first carry out an intraclass optimization by a local search, thereby continuously updating their own fitness values. In N iterations of local optimization, the quality of the whole frog population is optimized with the improvement of the quality of frogs in all groups. The frogs of the population are then fused and regrouped according to the established rule, and local optimization within the group is carried out until reaching the final iteration conditions. Finally, the global optimal solution of the frog population is defined as the optimal PCNN parameter configuration. The final fusion image is thus obtained using the optimal parameter configuration above.

## Results

In order to verify the accuracy and preservation of the edge details in our proposed method, three sets of CT and SPECT images were fused based on our method. The results of each set were compared with four fusion methods; IHS, NSCT+FL, DWT, NSCT+PCNN. In the method of NSCT+FL, images are first decomposed by NSCT to obtain high-frequency and low-frequency coefficients, and then fusion images are obtained by taking large value high-frequency coefficients and taking average value low-frequency coefficients. In NSCT+PCNN, images are decomposed by NSCT and fused by the PCNN.

### Subjective Evaluations of Experimental Results

Experiments were implemented on the image database from the Whole Brain Web Site of Harvard Medical School (Johnson and Becker, 2001) which contains two groups of images including CT and SPECT images. Each group has three examples including normal brain images, glioma brain images and brain images of patients diagnosed with Alzheimer’s disease. The testing images have been used in many related papers (Du et al., 2016a,b,c) and the platform is MATLAB R2018a.

**FIGURE 5:**
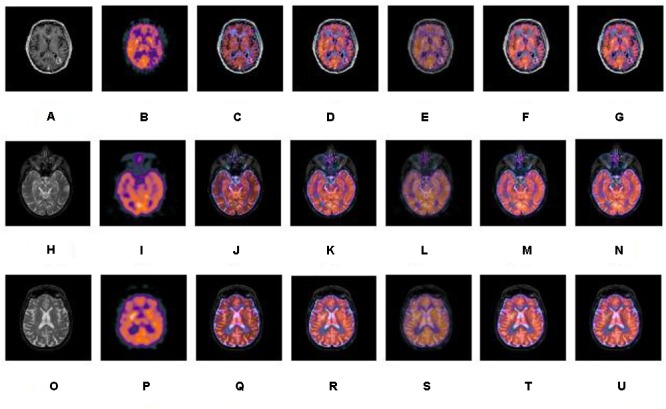
A series of contrasting experiments for normal brain images on fusion images based on different fusion methods (set 1). **(A,H,O)** are source CT images; **(B,I,P)** are source SPECT images; **(C,J,Q)** are fused images based on IHS; **(D,K,R)** are fused images based on NSCT+FL; **(E,L,S)** are fused images based on DWT; **(F,M,T)** are fused images based on the combination of NSCT+PCNN; **(G,N,U)** are fused images based on the proposed method.

**FIGURE 6:**
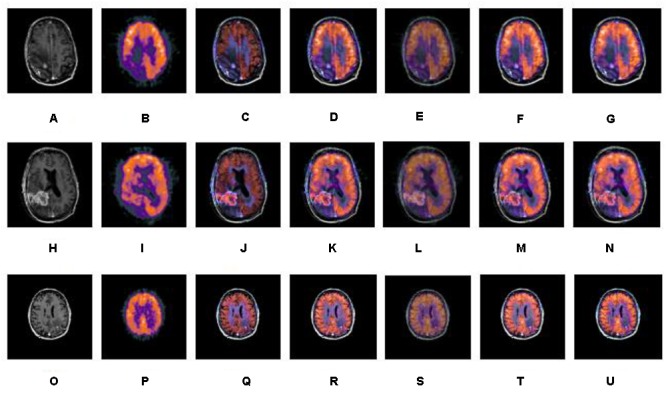
A series of contrasting experiments for glioma brain images on fusion images based on different fusion methods (set 2). **(A,H,O)** are source CT images; **(B,I,P)** are source SPECT images; **(C,J,Q)** are fused images based on IHS; **(D,K,R)** are fused images based on NSCT+FL; **(E,L,S)** are fused images based on DWT; **(F,M,T)** are fused images based on the combination of NSCT+PCNN; **(G,N,U)** are fused images based on the proposed method.

**FIGURE 7:**
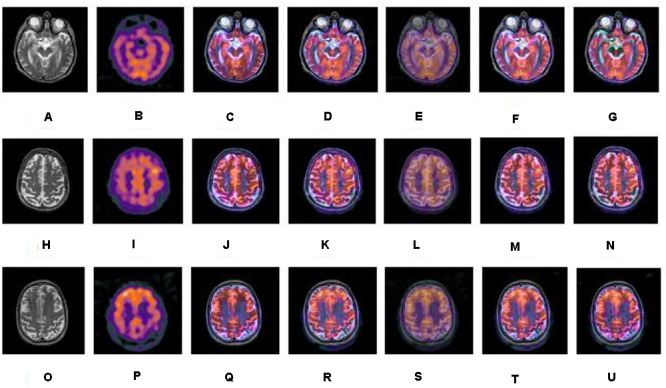
A series of contrasting experiments for brain images of patients diagnosed with Alzheimer’s disease on fusion images based on different fusion methods (set 3). **(A,H,O)** are source CT images; **(B,I,P)** are source SPECT images; **(C,J,Q)** are fused images based on IHS; **(D,K,R)** are fused images based on NSCT+FL; **(E,L,S)** are fused images based on DWT; **(F,M,T)** are fused images based on the combination of NSCT+PCNN; **(G,N,U)** are fused images based on the proposed method.

A series of fusion results of SPECT and CT images, based on different methods including IHS, NSCT+FL, DWT, NSCT+PCNN, and our proposed method is shown in Figure 5–7. The fusion results of a set of normal brain images are shown in Figure 5, the fusion results of a set of glioma brain images are presented in Figure 6, while a set of brain images of patients diagnosed with Alzheimer’s disease are shown in Figure 7. In Figure 5–7, (a), (h) and (o) are source CT images; (b), (i), (p) are source SPECT images; (c), (j) and (q) are fused images based on IHS; (d), (k) and (r) are fused images based on NSCT+FL; (e), (l) and (s) are fused images based on DWT; (f), (m) and (t) are fused images based on the combination of NSCT+PCNN; (g), (n) and (u) are fused images based on the proposed method. It can be seen that the fusion results based on our proposed method are more accurate and clearer than those based on various other methods. Our proposed method contributes to a higher brightness of fusion images and more information on the edge details.

### Objective Evaluations of Experimental Results

A set of metrics is used to compare the performance of the fusion methods including IHS, DWT, NSCT, PCNN, a combination of NSCT and the PCNN, and our proposed method. The evaluation metrics including standard deviation (SD), mean gradient (Ḡ), spatial frequency (SF) and information entropy (E) are entailed as follows (Huang et al., 2018):

(1) Standard deviation
Standard deviation is used to evaluate the contrast of the fused image, which is defined as

(2) $$\begin{array}{c} \sigma =\sqrt{\sum_{i=1}^{M}\sum_{j=1}^{N}(Z(i,j)-\bar{Z}{)}^{2}/(M\times N)} \end{array}$$

where Z(i,j) represents the pixel value of the fused image and $\bar{Z}$ is the mean value of the pixel values of the image.
The SD reflects the discrete image gray scale relative to the mean value of gray scale. And a higher value of SD demonstrates the performance of a fused image.
(2) Mean gradient (Ḡ)
Ḡ corresponds to the ability of a fused image to represent the contrast of tiny details sensitively. It can be mathematically described as

(3) $$\begin{array}{c} \bar{G}=\frac{1}{\left(M-1)(N-1\right)}\sum_{i=1}^{M-1}\sum_{j=1}^{N-1}\times \sqrt{((\frac{\partial Z(x_{i},)}{\partial x_{i}}{)}^{2}+(\frac{\partial Z(x_{i},y_{j})}{\partial y_{i}}{)}^{2})/2} \end{array}$$

The fused image is clearer when the value of mean gradient is higher.
(3) Spatial frequency (SF)
Spatial frequency is the measure of the overall activity in a fused image. For an image with a gray value Z(x_i_,y_j_) at position (x_i_,y_j_), the spatial frequency is defined as

(4) $$\begin{array}{c} SF=\sqrt{RF^{2}+CF^{2}} \end{array}$$

Where row frequency

(5) $$\begin{array}{c} RF=\sqrt{\frac{1}{M\times N}\sum_{i=1}^{M}\sum_{j=2}^{N}{\left[Z(x_{i},\;y_{j})-Z(x_{i},y_{j-1})\right]}^{2}} \end{array}$$

Column frequency

(6) $$\begin{array}{c} CF=\sqrt{\frac{1}{M\times N}\sum_{i=2}^{M}\sum_{j=1}^{N}{\left[Z(x_{i},\;y_{j})-Z(x_{i-1},y_{j})\right]}^{2}} \end{array}$$

The higher the value of frequency, the better the fused image quality.
(4) Information entropy (E)
Information entropy is provided by the below equation

(7) $$\begin{array}{c} E=-\sum_{i=0}^{L-1}p_{i}\log_{2}p_{i} \end{array}$$

where L is image gray scale and Pi is the proportion of the pixel of the gray value i in whole pixels. A higher value of entropy indicates more information contained in the fused image.

Experiment results on fused images of SPECT images and CT images are shown in Tables 1–3. The fusion results of a set of normal brain images are shown in Table 1, the fusion results of a set of glioma brain images are presented in Table 2, while a set of brain images of patients diagnosed with Alzheimer’s disease are shown Table 3. It can be seen that compared to other fusion methods, our proposed method generally has higher values in SD, Ḡ, SF and E. The experimental results demonstrate that information of fusion images obtained by our proposed method is more abundant, the inheritance of detail information performs better, while the resolution is significantly improved.

**Table 1:** Performance evaluations on normal brain fused images based on different methods.

	Metric	IHS	NSCT+FL	DWT	NSCT+PCNN	Proposed
Set 1	Standard deviation	51.6141	55.2178	42.5312	57.1188	57.2258
	Mean gradient	8.8561	8.714	6.2027	8.8568	8.8071
	Spatial frequency	33.5851	33.2324	22.0093	33.7566	33.6546
	Information entropy	2.6859	2.7565	3.0483	2.7729	3.0621
Set 2	Standard deviation	43.278	49.5989	43.0915	52.9246	53.1691
	Mean gradient	6.686	6.6633	4.5622	6.5672	6.7489
	Spatial frequency	20.3855	19.9558	12.7416	19.8214	20.0956
	Information entropy	3.6325	3.9243	4.2501	3.8386	3.9424
Set 3	Standard deviation	50.0926	55.7124	47.4476	57.1246	57.1268
	Mean gradient	6.2153	6.1775	4.1822	6.086	6.1796
	Spatial frequency	19.244	18.9682	12.0096	18.7269	18.7335
	Information entropy	3.6226	3.7122	4.0074	3.7139	3.7399

**Table 2:** Performance evaluations on glioma brain fused images based on different methods.

	Metric	IHS	NSCT+FL	DWT	NSCT+PCNN	Proposed
Set 1	Standard deviation	41.7514	55.2055	39.8132	58.0374	58.3122
	Mean gradient	5.2953	5.5442	3.8166	5.459	5.5678
	Spatial frequency	16.2064	16.5277	10.1649	16.466	16.4776
	Information entropy	3.9255	4.1433	4.6303	4.08	4.1788
Set 2	Standard deviation	44.154	55.5879	42.436	57.7284	57.775
	Mean gradient	6.2881	6.6316	4.595	6.535	6.7276
	Spatial frequency	17.6675	17.9369	11.359	17.9359	17.9095
	Information entropy	4.3966	4.7513	5.1901	4.6312	4.837
Set 3	Standard deviation	48.6572	54.0708	41.78	56.2065	56.3546
	Mean gradient	6.8855	6.8515	4.8166	6.774	6.7977
	Spatial frequency	27.8964	27.8583	17.8725	27.7365	27.7654
	Information entropy	2.4852	2.5749	2.8442	2.5239	2.658

**Table 3:** Performance evaluations on fused brain images of patients diagnosed with Alzheimer’s disease, based on different methods.

	Metric	IHS	NSCT+FL	DWT	NSCT+PCNN	Proposed
Set 1	Standard deviation	66.1357	65.3766	51.0336	69.5392	66.5782
	Mean gradient	9.9938	10.0303	6.509	10.0089	10.2068
	Spatial frequency	26.7087	26.7329	16.1614	26.6568	27.1771
	Information entropy	4.7735	4.834	5.4105	4.8036	4.8966
Set 2	Standard deviation	59.1931	59.2093	52.0837	61.4981	60.6457
	Mean gradient	6.7482	7.0266	4.5756	7	7.0461
	Spatial frequency	19.0263	19.3264	11.8249	19.3257	19.512
	Information entropy	3.9901	4.1834	4.5922	4.0985	4.2156
Set 3	Standard deviation	56.0974	58.787	47.6032	56.0943	57.7578
	Mean gradient	7.9023	8.111	5.4579	7.9592	7.966
	Spatial frequency	22.2846	22.4084	13.907	21.9421	22.0022
	Information entropy	3.895	4.1058	5.1943	4.2228	4.2897

## Conclusion

In this paper, a new fusion method for SPECT brain and CT brain images was put forward. First, NSCT was used to decompose the IHS transform of a SPECT and CT image. The fusion rules, based on the regional average energy, was then used for low-frequency coefficients and the combination of SFLA and the PCNN was used for high-frequency sub-bands. Finally, the fused image was produced by reversed NSCT and reversed IHS transform. Both subjective evaluations and objective evaluations were used to analyze the quality of the fused images. The results demonstrated that the method we put forward can retain the information of source images better and reveal more details in integration. It can be seen that the proposed method is valid and effective in achieving satisfactory fusion results, leading to a wide range of applications in practice.

The paper focuses on multi-mode medical image fusion. However, there is a negative correlation between the real-time processing speed and the effectiveness of medical image fusion. Under the premise of ensuring the quality of fusion results, how to improve the efficiency of the method should be considered in the future.

## Data Availability

Publicly available datasets were analyzed in this study. This data can be found here: http://www.med.harvard.edu/aanlib/.

## Author Contributions

CH conceived the study. GT and CH designed the model. YC and YP analyzed the data. YL and WC wrote the draft. EN and YH interpreted the results. All authors gave critical revision and consent for this submission.

## Conflict of Interest Statement

The authors declare that the research was conducted in the absence of any commercial or financial relationships that could be construed as a potential conflict of interest.
